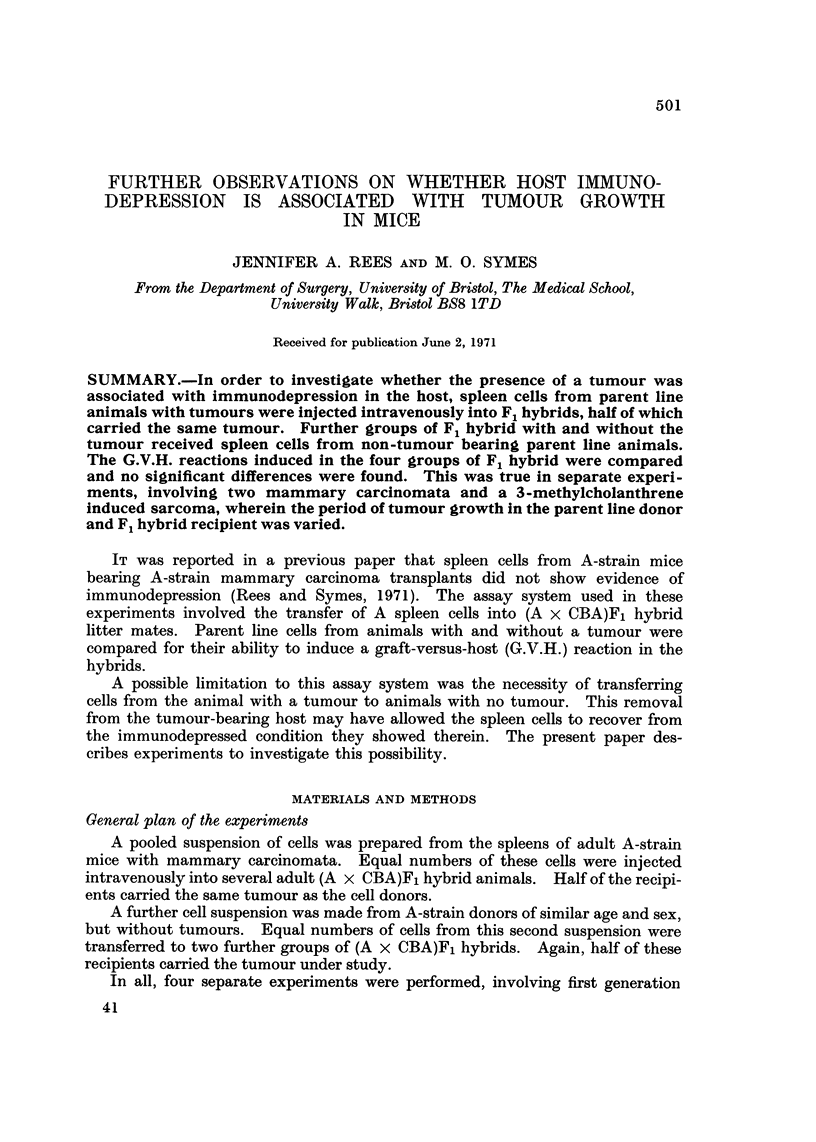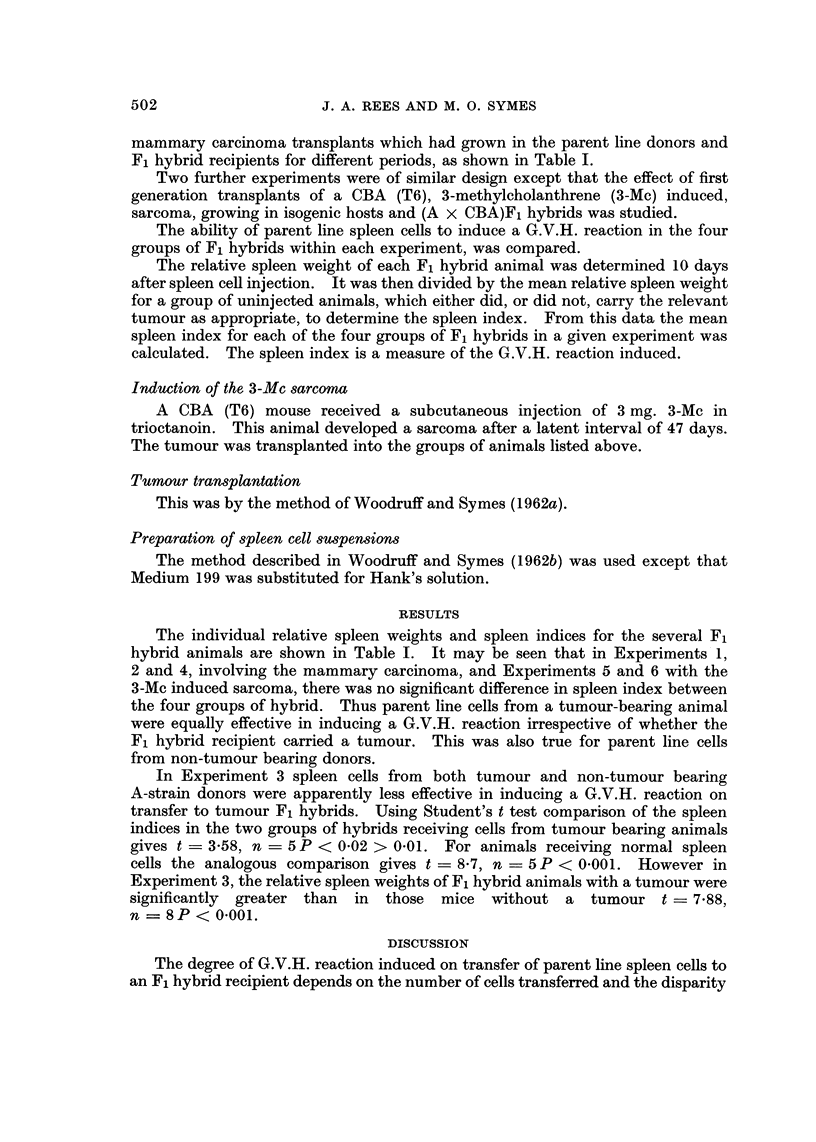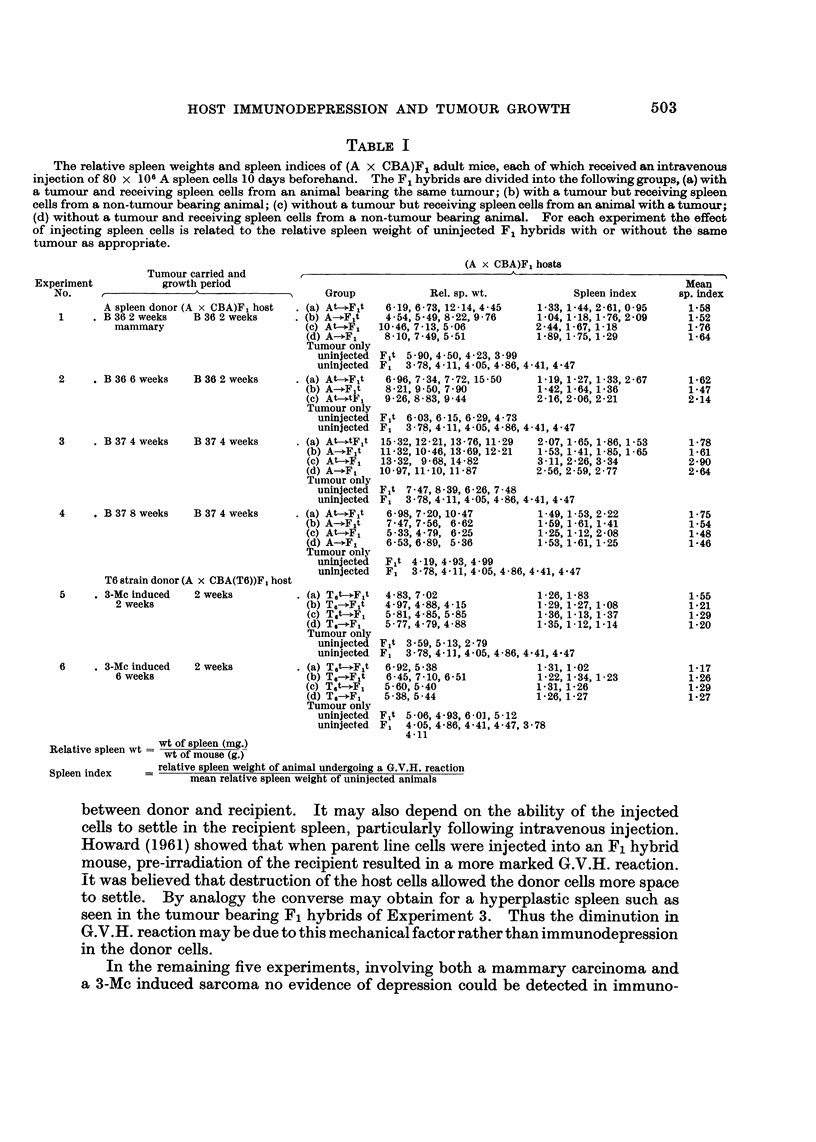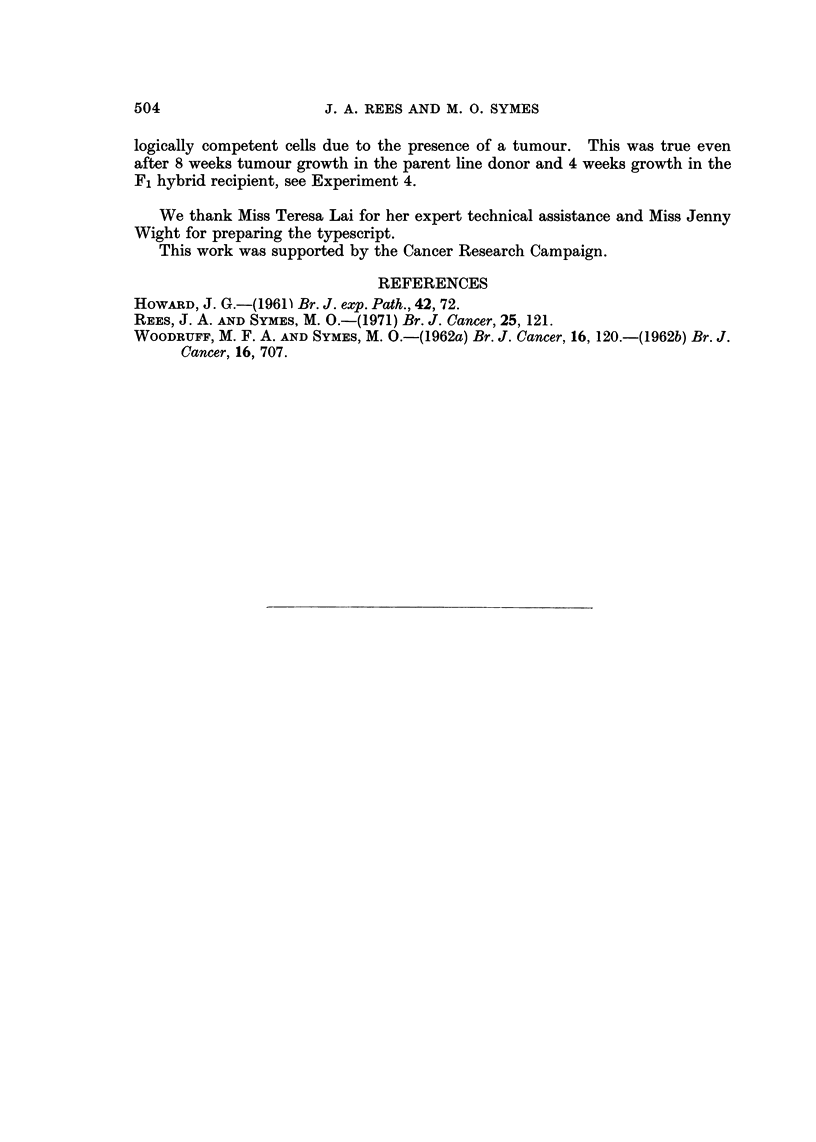# Further Observations on Whether Host Immunodepression is Associated with Tumour Growth in Mice

**DOI:** 10.1038/bjc.1971.63

**Published:** 1971-09

**Authors:** Jennifer A. Rees, M. O. Symes

## Abstract

In order to investigate whether the presence of a tumour was associated with immunodepression in the host, spleen cells from parent line animals with tumours were injected intravenously into F_1_ hybrids, half of which carried the same tumour. Further groups of F_1_ hybrid with and without the tumour received spleen cells from non-tumour bearing parent line animals. The G.V.H. reactions induced in the four groups of F_1_ hybrid were compared and no significant differences were found. This was true in separate experiments, involving two mammary carcinomata and a 3-methylcholanthrene induced sarcoma, wherein the period of tumour growth in the parent line donor and F_1_ hybrid recipient was varied.


### FURTHER OBSERVATIONS ON WHETHER HOST IMMUNODEPRESSION IS ASSOCIATED WITH TUMOUR GROWTH IN MICE

JENNIFER A. REES AND M. O. SYMES

*From the Department of Surgery, University of Bristol, The Medical School, University Walk, Bristol BS8 1TD*

Received for publication June 2, 1971

**SUMMARY.—In order to investigate whether the presence of a tumour was associated with immunodepression in the host, spleen cells from parent line animals with tumours were injected intravenously into $F_1$ hybrids, half of which carried the same tumour. Further groups of $F_1$ hybrid with and without the tumour received spleen cells from non-tumour bearing parent line animals. The G.V.H. reactions induced in the four groups of $F_1$ hybrid were compared and no significant differences were found. This was true in separate experiments, involving two mammary carcinomata and a 3-methylcholanthrene induced sarcoma, wherein the period of tumour growth in the parent line donor and $F_1$ hybrid recipient was varied.**

It was reported in a previous paper that spleen cells from A-strain mice bearing A-strain mammary carcinoma transplants did not show evidence of immunodepression (Rees and Symes, 1971). The assay system used in these experiments involved the transfer of A spleen cells into (A × CBA)$F_1$ hybrid litter mates. Parent line cells from animals with and without a tumour were compared for their ability to induce a graft-versus-host (G.V.H.) reaction in the hybrids.

A possible limitation to this assay system was the necessity of transferring cells from the animal with a tumour to animals with no tumour. This removal from the tumour-bearing host may have allowed the spleen cells to recover from the immunodepressed condition they showed therein. The present paper describes experiments to investigate this possibility.

### MATERIALS AND METHODS

### *General plan of the experiments*

A pooled suspension of cells was prepared from the spleens of adult A-strain mice with mammary carcinomata. Equal numbers of these cells were injected intravenously into several adult (A × CBA)$F_1$ hybrid animals. Half of the recipients carried the same tumour as the cell donors.

A further cell suspension was made from A-strain donors of similar age and sex, but without tumours. Equal numbers of cells from this second suspension were transferred to two further groups of (A × CBA)$F_1$ hybrids. Again, half of these recipients carried the tumour under study.

In all, four separate experiments were performed, involving first generation mammary carcinoma transplants which had grown in the parent line donors and $F_1$ hybrid recipients for different periods, as shown in Table I.

Two further experiments were of similar design except that the effect of first generation transplants of a CBA (T6), 3-methylcholanthrene (3-Mc) induced, sarcoma, growing in isogenic hosts and (A $\times$ CBA)$F_1$ hybrids was studied.

The ability of parent line spleen cells to induce a G.V.H. reaction in the four groups of $F_1$ hybrids within each experiment, was compared.

The relative spleen weight of each $F_1$ hybrid animal was determined 10 days after spleen cell injection. It was then divided by the mean relative spleen weight for a group of uninjected animals, which either did, or did not, carry the relevant tumour as appropriate, to determine the spleen index. From this data the mean spleen index for each of the four groups of $F_1$ hybrids in a given experiment was calculated. The spleen index is a measure of the G.V.H. reaction induced.

*Induction of the* 3-*Mc sarcoma*

A CBA (T6) mouse received a subcutaneous injection of 3 mg. 3-Mc in trioctanoin. This animal developed a sarcoma after a latent interval of 47 days. The tumour was transplanted into the groups of animals listed above.

*Tumour transplantation*

This was by the method of Woodruff and Symes (1962*a*).

*Preparation of spleen cell suspensions*

The method described in Woodruff and Symes (1962*b*) was used except that Medium 199 was substituted for Hank's solution.

### RESULTS

The individual relative spleen weights and spleen indices for the several $F_1$ hybrid animals are shown in Table I. It may be seen that in Experiments 1, 2 and 4, involving the mammary carcinoma, and Experiments 5 and 6 with the 3-Mc induced sarcoma, there was no significant difference in spleen index between the four groups of hybrid. Thus parent line cells from a tumour-bearing animal were equally effective in inducing a G.V.H. reaction irrespective of whether the $F_1$ hybrid recipient carried a tumour. This was also true for parent line cells from non-tumour bearing donors.

In Experiment 3 spleen cells from both tumour and non-tumour bearing A-strain donors were apparently less effective in inducing a G.V.H. reaction on transfer to tumour $F_1$ hybrids. Using Student's *t* test comparison of the spleen indices in the two groups of hybrids receiving cells from tumour bearing animals gives $t = 3{\cdot}58$, $n = 5\,P < 0{\cdot}02 > 0{\cdot}01$. For animals receiving normal spleen cells the analogous comparison gives $t = 8{\cdot}7$, $n = 5\,P < 0{\cdot}001$. However in Experiment 3, the relative spleen weights of $F_1$ hybrid animals with a tumour were significantly greater than in those mice without a tumour $t = 7{\cdot}88$, $n = 8\,P < 0{\cdot}001$.

### DISCUSSION

The degree of G.V.H. reaction induced on transfer of parent line spleen cells to an $F_1$ hybrid recipient depends on the number of cells transferred and the disparity

### Table I

The relative spleen weights and spleen indices of (A × CBA)$F_1$ adult mice, each of which received an intravenous injection of 80 × $10^6$ A spleen cells 10 days beforehand. The $F_1$ hybrids are divided into the following groups, (a) with a tumour and receiving spleen cells from an animal bearing the same tumour; (b) with a tumour but receiving spleen cells from a non-tumour bearing animal; (c) without a tumour but receiving spleen cells from an animal with a tumour; (d) without a tumour and receiving spleen cells from a non-tumour bearing animal. For each experiment the effect of injecting spleen cells is related to the relative spleen weight of uninjected $F_1$ hybrids with or without the same tumour as appropriate.

| Experiment No. | Tumour carried and growth period | | (A × CBA)$F_1$ hosts | | | |
|---|---|---|---|---|---|---|
| | A spleen donor | (A × CBA)$F_1$ host | Group | Rel. sp. wt. | Spleen index | Mean sp. index |
| 1 | B 36 2 weeks mammary | B 36 2 weeks | (a) At→$F_1$t | 6·19, 6·73, 12·14, 4·45 | 1·33, 1·44, 2·61, 0·95 | 1·58 |
| | | | (b) A→$F_1$t | 4·54, 5·49, 8·22, 9·76 | 1·04, 1·18, 1·76, 2·09 | 1·52 |
| | | | (c) At→$F_1$ | 10·46, 7·13, 5·06 | 2·44, 1·67, 1·18 | 1·76 |
| | | | (d) A→$F_1$ | 8·10, 7·49, 5·51 | 1·89, 1·75, 1·29 | 1·64 |
| | | | Tumour only uninjected | $F_1$t 5·90, 4·50, 4·23, 3·99 | | |
| | | | uninjected | $F_1$ 3·78, 4·11, 4·05, 4·86, 4·41, 4·47 | | |
| 2 | B 36 6 weeks | B 36 2 weeks | (a) At→$F_1$t | 6·96, 7·34, 7·72, 15·50 | 1·19, 1·27, 1·33, 2·67 | 1·62 |
| | | | (b) A→$F_1$t | 8·21, 9·50, 7·90 | 1·42, 1·64, 1·36 | 1·47 |
| | | | (c) At→t$F_1$ | 9·26, 8·83, 9·44 | 2·16, 2·06, 2·21 | 2·14 |
| | | | Tumour only uninjected | $F_1$t 6·03, 6·15, 6·29, 4·73 | | |
| | | | uninjected | $F_1$ 3·78, 4·11, 4·05, 4·86, 4·41, 4·47 | | |
| 3 | B 37 4 weeks | B 37 4 weeks | (a) At→t$F_1$t | 15·32, 12·21, 13·76, 11·29 | 2·07, 1·65, 1·86, 1·53 | 1·78 |
| | | | (b) A→$F_1$t | 11·32, 10·46, 13·69, 12·21 | 1·53, 1·41, 1·85, 1·65 | 1·61 |
| | | | (c) At→$F_1$ | 13·32, 9·68, 14·82 | 3·11, 2·26, 3·34 | 2·90 |
| | | | (d) A→$F_1$ | 10·97, 11·10, 11·87 | 2·56, 2·59, 2·77 | 2·64 |
| | | | Tumour only uninjected | $F_1$t 7·47, 8·39, 6·26, 7·48 | | |
| | | | uninjected | $F_1$ 3·78, 4·11, 4·05, 4·86, 4·41, 4·47 | | |
| 4 | B 37 8 weeks | B 37 4 weeks | (a) At→$F_1$t | 6·98, 7·20, 10·47 | 1·49, 1·53, 2·22 | 1·75 |
| | | | (b) A→$F_1$t | 7·47, 7·56, 6·62 | 1·59, 1·61, 1·41 | 1·54 |
| | | | (c) At→$F_1$ | 5·33, 4·79, 6·25 | 1·25, 1·12, 2·08 | 1·48 |
| | | | (d) A→$F_1$ | 6·53, 6·89, 5·36 | 1·53, 1·61, 1·25 | 1·46 |
| | | | Tumour only uninjected | $F_1$t 4·19, 4·93, 4·99 | | |
| | | | uninjected | $F_1$ 3·78, 4·11, 4·05, 4·86, 4·41, 4·47 | | |
| | T6 strain donor | (A × CBA(T6))$F_1$ host | | | | |
| 5 | 3-Mc induced 2 weeks | 2 weeks | (a) $T_6$t→$F_1$t | 4·83, 7·02 | 1·26, 1·83 | 1·55 |
| | | | (b) $T_6$→$F_1$t | 4·97, 4·88, 4·15 | 1·29, 1·27, 1·08 | 1·21 |
| | | | (c) $T_6$t→$F_1$ | 5·81, 4·85, 5·85 | 1·36, 1·13, 1·37 | 1·29 |
| | | | (d) $T_6$→$F_1$ | 5·77, 4·79, 4·88 | 1·35, 1·12, 1·14 | 1·20 |
| | | | Tumour only uninjected | $F_1$t 3·59, 5·13, 2·79 | | |
| | | | uninjected | $F_1$ 3·78, 4·11, 4·05, 4·86, 4·41, 4·47 | | |
| 6 | 3-Mc induced 6 weeks | 2 weeks | (a) $T_6$t→$F_1$t | 6·92, 5·38 | 1·31, 1·02 | 1·17 |
| | | | (b) $T_6$→$F_1$t | 6·45, 7·10, 6·51 | 1·22, 1·34, 1·23 | 1·26 |
| | | | (c) $T_6$t→$F_1$ | 5·60, 5·40 | 1·31, 1·26 | 1·29 |
| | | | (d) $T_6$→$F_1$ | 5·38, 5·44 | 1·26, 1·27 | 1·27 |
| | | | Tumour only uninjected | $F_1$t 5·06, 4·93, 6·01, 5·12 | | |
| | | | uninjected | $F_1$ 4·05, 4·86, 4·41, 4·47, 3·78, 4·11 | | |

$$\text{Relative spleen wt} = \frac{\text{wt of spleen (mg.)}}{\text{wt of mouse (g.)}}$$

$$\text{Spleen index} = \frac{\text{relative spleen weight of animal undergoing a G.V.H. reaction}}{\text{mean relative spleen weight of uninjected animals}}$$

between donor and recipient. It may also depend on the ability of the injected cells to settle in the recipient spleen, particularly following intravenous injection. Howard (1961) showed that when parent line cells were injected into an $F_1$ hybrid mouse, pre-irradiation of the recipient resulted in a more marked G.V.H. reaction. It was believed that destruction of the host cells allowed the donor cells more space to settle. By analogy the converse may obtain for a hyperplastic spleen such as seen in the tumour bearing $F_1$ hybrids of Experiment 3. Thus the diminution in G.V.H. reaction may be due to this mechanical factor rather than immunodepression in the donor cells.

In the remaining five experiments, involving both a mammary carcinoma and a 3-Mc induced sarcoma no evidence of depression could be detected in immunologically competent cells due to the presence of a tumour. This was true even after 8 weeks tumour growth in the parent line donor and 4 weeks growth in the $F_1$ hybrid recipient, see Experiment 4.

We thank Miss Teresa Lai for her expert technical assistance and Miss Jenny Wight for preparing the typescript.

This work was supported by the Cancer Research Campaign.

### REFERENCES

HOWARD, J. G.—(1961) *Br. J. exp. Path.*, **42**, 72.

REES, J. A. AND SYMES, M. O.—(1971) *Br. J. Cancer*, **25**, 121.

WOODRUFF, M. F. A. AND SYMES, M. O.—(1962*a*) *Br. J. Cancer*, **16**, 120.—(1962*b*) *Br. J. Cancer*, **16**, 707.